# COVID-19 and British Columbia’s volunteer search and rescue workers: risk recognition and risk mitigation

**DOI:** 10.1017/ash.2023.463

**Published:** 2023-11-03

**Authors:** David Birnbaum, Vienna C. Lam, Farinaz Havaei, Gail S. Anderson

**Affiliations:** 1 Applied Epidemiology, North Saanich, BC, USA; 2 The University of British Columbia School of Population & Public Health, Vancouver, BC, USA; 3 Centre for Forensic Research, School of Criminology, Simon Fraser University, Burnaby, BC, USA; 4 University of British Columbia School of Nursing, Vancouver, BC, USA

## Abstract

**Background:**

Early during COVID-19, British Columbia coordinated collaboration between academic researchers, public healthcare systems, and private sector partners to focus research resources on knowledge gaps in a timely manner, avoid duplication, and identify overlooked aspects. At a collaboration symposium, it became evident that BC’s volunteer search & rescue (SAR) cadre was overlooked.

**Objective:**

Our exploratory project studied volunteer SAR’s operational readiness; use and perceived value of information sources; consistency in infection prevention measures among volunteer stations, and with their professional counterparts for comparable first aid medical interventions throughout the pandemic.

**Methods:**

We partnered with the 2 organizations that govern BC’s volunteer SAR stations. Local station leaders completed a short confidential survey. Guidance documents issued by associations governing voluntary and professional first responders were compared.

**Results:**

Survey responses were received from 33 of 109 local stations, spanning all regions of BC. Most remained operationally ready throughout the entire pandemic (12.1% had to stand down at times). Except for 21% lacking eye protection, all had personal protective equipment commensurate with that of healthcare professionals; however, few used this PPE in a manner consistent with professional counterparts. Usage and perceived usefulness of various information sources differed. There was no communication link between the province’s infection control experts and 2 volunteer SAR organizations.

**Conclusions:**

Search & rescue capability was maintained despite pandemic impacts. Results reveal strengths and opportunities for improvement in the ways volunteers are informed and protected. Infection control experts providing advice for emergency health services professional responders should remember to include their volunteer counterparts.

## Introduction

Early in the COVID-19 pandemic, British Columbia coordinated collaborative efforts between its academic researchers, public healthcare systems, and private sector partners to focus research resources on knowledge gaps in a timely manner, avoid duplication of effort, and identify any overlooked aspects. During its September 2020 free public BC COVID-19 Research and Collaboration Symposium, it became evident that no research had been initiated to understand impact on BC’s volunteer search & rescue (SAR) cadre.^1^ Despite being an extension of BC’s emergency services, it is possible that volunteer SAR first responders did not receive timely updates and communications regarding COVID-19 infection prevention for risk recognition and mitigation during first-aid procedures commensurate to their professional counterparts for self-protection during comparable medical interventions. The implication is not that volunteer SAR members face identical frequency or magnitude of COVID-19 infection risk as healthcare professionals inside hospitals; rather, it is that there should be consistency in precautions taken during comparable risk exposure procedures. For example, resuscitating drowning victims is comparable to the aerosol-generating hospital procedure of nebulized sputum induction; administering high-flow supplemental oxygen (whether during first aid in rescue or medical care inside hospitals) has been designated as a possible aerosol-generating procedure.^2^

Approximately 3,500 individuals volunteer on BC’s SAR crews. Volunteer marine SAR is governed by the Royal Canadian Marine Search and Rescue (RCMSAR), organized in 31 local stations around the province (https://ccga-pacific.org/about.php). Ground-based SAR is governed by the British Columbia Search and Rescue Association (BCSARA), organized in 78 local stations (https://www.bcsara.com/about/sar-in-bc/). Local station volunteers, the first link in continuity of care, often respond to prevent loss of life, rendering first aid in emergency situations in support of professional Coast Guard, paramedic, police, or firefighter first responders. Trained volunteers can often be the first on scene or the only responding unit. These ordinary citizens, from all backgrounds, ages 20s through 70s, are tasked out in teams by BC’s Rescue Coordination Centre (https://www.canada.ca/en/department-national-defence/services/operations/military-operations/types/search-rescue/western-canada.html) on thousands of missions each year to locate, stabilize, and rapidly transport people in distress to higher levels of care. Stations are primarily self-funded (charitable grants & donations), government-reimbursed only for fuel-type supplies during missions.

British Columbia Search and Rescue Association and RCMSAR kept their local stations informed by periodically sending information and instructions to station leaders throughout this pandemic, and posting such documents in member-only website sections. Local stations may have supplemented this with other sources of timely information related to the evolving COVID-19 situation. The extent to which this optimized risk recognition and mitigation practices within local stations, or resulted in consistency of measures across stations, has not previously been assessed by either organization and until now wasn’t addressed by BC’s academic research community. Whether volunteer SAR is an overlooked sector; current evidence-informed infection control measures during aerosol-generating procedures in hospitals differ from commensurate practices among first responders; different SAR units within marine and ground-based organizations have been kept advised in different ways and whether that led to inconsistencies in practices; whether inconsistencies might leave some units at greater risk than others; and in what ways COVID-19 impacted the health and operational readiness of SAR units, are pertinent questions.

## Methods

This project had 3 phases: (1) survey of local stations, (2) review of official update documents provided to them throughout the pandemic by BCSARA and RCMSAR independent of each other, and (3) comparison of advice and practices within this volunteer SAR community versus concurrent advice and practices among their professional first responder and healthcare professional counterparts. The research protocol was approved under UBC Research Ethics Board certificate H21-00757.

Our literature search failed to find any previous work of this nature, so a survey questionnaire was devised by one of the authors experienced both as a hospital epidemiologist and volunteer SAR crew member (DB). Initial drafts were reviewed by all members of the academic team for refinement, then shared with officers of our community partners (BCSARA & RCMSAR) to confirm face and content validity. End result was a combination of open-ended, interval Likert-scale, and ratio-scale questions. The form contained three questions regarding what sources of information stations found useful, one regarding what personal protective equipment (PPE) items they’d used and how acquired, and three regarding extent to which COVID-19 impacted ability to maintain operational readiness.

In March 2022, a questionnaire and cover letter requesting voluntary participation was emailed by RCMSAR to all its 31 station leaders and by BCSARA to all its 78 local station leaders across the province. A reminder prompt to respond was emailed midway through the 2–4 week request deadline period. Application of the Finite Population Correction Factor^3^ to statistical power calculation set 44 for BCSARA & 23 for RCMSAR as maximum sample size, 15 & 12 as respective minimums, required to estimate true proportions within ±10% at a 95% confidence level. Surveys were completed and returned by station leaders via email to the study’s academic research lead, compiled after removing personal identifying information, and descriptive summary statistics preserving confidentiality were generated using IBM SPSS version 28.0.0.0 (190). Bonferroni correction was applied for multiple comparisons.^3^

Update documents independently issued by BCSARA, RCMSAR, and their professional counterparts (emergency health services’ firefighter or police first responders and paramedics) were obtained from those organizations, compared in side-by-side reading with each other and what station leaders indicated had or had not been used locally.

## Results

### Survey response rate & pattern

Survey responses were provided by 10 of the 31 RCMSAR and 23 of the 78 BCSARA local stations (overall 30.3% response rate). The province is divided into 6 regions by Emergency Management BC (see map, https://www2.gov.bc.ca/assets/gov/public-safety-and-emergency-services/emergency-preparedness-response-recovery/embc/embc_regional_office_map_by_regional_district.pdf). Responses were received from local stations in large urban and smaller rural locations throughout all six regions.

### Survey answers

Except for eye protection (face shield or safety goggles), all had PPE protective against all routes of COVID-19 transmission initially thought possible (Tables 1–3). However, volunteers differed from professionals in mask selection criteria (Tables 4 and 5). Also, not all SAR stations have replaced latex with nitrile gloves, unlike hospitals, which started replacing latex as an allergy safety issue about 25 years ago.

**Table 1.** Proportion of stations equipped with personal protective equipment (PPE) items and how they were procured

Item	BCSARA	RCMSAR
Eye protection Donated Headquarter supplied Local purchase Member’s own	Present in 20 of 23 35.0% (7/20) 15.0% (3/20) 65.0% (13/20) 25.0% (5/20)	Present in 6 of 10 50.0% (3/6) 16.7% (1/6) 50.0% (3/6) 0.0% (0/6)
Respiratory protection (mask) *Donated* *Headquarter supplied* *Local purchase* *Member’s own*	Present in 23 of 23 40.0% (17/46) 73.9% (34/46) 58.7% (27/46) 32.6% (15/46)	Present in 10 of 10 15.0% (3/20) 15.0% (3/20) 65.0%% (13/20) 0.0% (0/20)
Skin protection (gloves) *Donated* *Headquarter supplied* *Local purchase* *Member’s own*	Present in 23 of 23 8.7% (4/46) 10.9% (5/46) 69.6% (32/46) 13.0% (6/46)	Present in 10 of 10 15.0% (3/20) 5.0% (1/20) 70.0% (14/20) 0.0% (0/20)
Hand sanitizer *Donated* *Headquarter supplied* *Local purchase* *Member’s own*	Present in 23 of 23 43.5% (10/23) 26.1% (6/23) 78.3% (18/23) 21.7% (5/23)	Present in 10 of 10 30.0% (3/10) 20.0% (2/10) 70.0% (7/10) 0.0% (0/10)
Surface disinfectant *Donated* *Headquarter supplied* *Local purchase* *Member’s own*	Present in 23 of 23 21.7% (5/23) 4.3% (1/23) 91.3% (21/23) 0.0% (0/23)	Present in 10 of 10 20.0% (2/10) 0.0% (0/10) 90.0% (9/10) 0.0% (0/10)

Note. Percentages total more than 100 because many stations used more than 1 source of acquisition to procure any given item type. Denominators are doubled for respiratory protection and skin protection because these items’ numerators are tallied from 2 separate variables (surgical & N95 masks; latex & nitrile gloves).

**Table 2.** Extent to which stations had surgical masks and/or N95 respirators

			N95		
			No	Yes	Row Total
BCSARA	Surgical	No	0	0	0
		Yes	0	23	23
	Column subtotal		0	23	23
RCMSAR	Surgical	No	0	0	0
		Yes	3	7	10
	Column subtotal		3	7	10

**Table 3.** Extent to which stations had latex and/or nitrile gloves

			Nitrile		
			No	Yes	Row Total
BCSARA	Latex	No	0	8	8
		Yes	2	13	15
	Column subtotal		2	21	23
RCMSAR	Latex	No	0	0	0
		Yes	2	8	10
	Colum subtotal		2	8	10

**Table 4.** Criterion for selecting to wear surgical mask vs. N95 respirator

		BCSARA	RCMSAR	Overall
Surgical vs. N95 Mask Selection criterion	Personal preference	11	3	14
	Proximity to crew vs. public	2	2	4
	Other	3	1	4
	Suspected or known COVID-19 case	3	0	3
	No N95 available	0	3	3
	Surgical until N95 is widely available	2	1	3
	Aerosol generating procedure	2	0	2
Total		23	10	33

**Table 5.** Criteria among stations that reported having both surgical & N95 masks

Volunteers in BCSARA & RCMSAR	Healthcare Professionals in BC Hospitals
Individual personal preferences (47%); Surgical for proximity to crew, N95 for proximity to public (13%); Location (13%) - N95 inside (vehicle, training or command unit), surgical outside (6%); - N95 available but not used (3%); - Unspecified/unclear (3%); N95 if suspected or known COVID-19 patient (10%); Surgical until N95 became widely available (10%); Just 7% identified switching from surgical to N95 if performing aerosol-generating procedures.	Surgical mask sufficient for most patient encounters, N95 instead during aerosol-generating procedures (100%)

**Figure 1.**
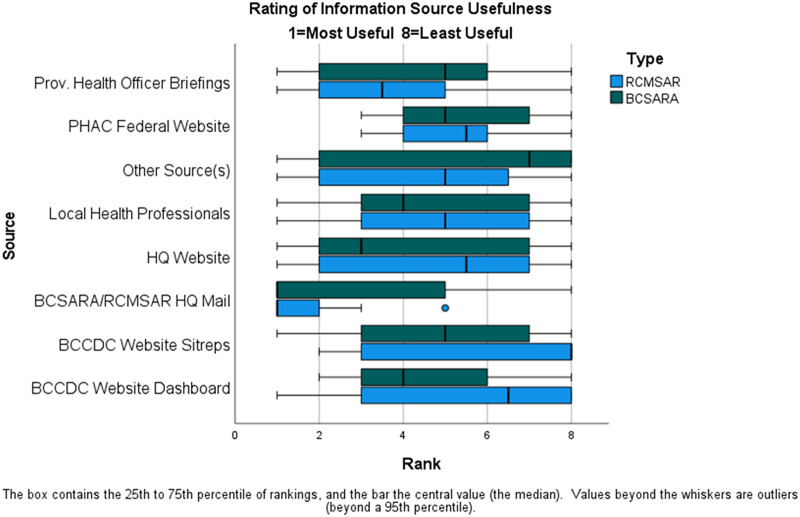
Perceived utility of various information sources.

Beyond BCSARA and RCMSAR, COVID-19 information was available from the Provincial Health Officer, the BC Centre for Disease Control (BCCDC), the Public Health Agency of Canada (PHAC), and elsewhere (Figure 1). Differences in distribution of rankings by BCSARA versus RCMSAR (Figure 1) are not statistically significant (*p* > 0.05, normal approximation to Wilcoxon rank sum test with Bonferroni and Finite Population Correction Factors applied).^3,4^ Reasons given for considering some more helpful than others include being issued in sufficiently timely manner, containing up-to-date information and coming from a reputable source; clarity; having content specific to SAR activities (although one commented it was not clear whether documents labelled as advisories were mandatory or simply recommendations); and ease of access (“push” modalities like emailed updates and television broadcasts favored over “pull” modalities like having to visit websites). Some commented that monitoring websites is not only time-consuming but also could be subject to individual interpretations. Headquarter mailings were, by far, the source most frequently reported by station leaders and always shared with their members. Update information from health officers was also watched less consistently but still relatively frequently. Health officer updates broadcast often (relating weekly case counts and trends, periodically announcing new precautions), while headquarter update frequency was intermittent (issued only when it was deemed necessary to announce changes in precautionary SAR practices), so it is not surprising that more respondents reported sharing all headquarters updates with their members versus sharing health officer information only if it contained important changes.

Among the 33 responses, 20 provided a usefulness ranking for “other” source(s) of guidance (the other 13 left that line blank). Within those 20, 8 ranked “other” sources as highly useful (scoring as 1–3); 6 of the 8 identified use of news media, local healthcare professionals or their regional health authority, an epidemiologist, or foreign websites (CDC, WHO); the remaining 2 did not identify any “other” source as having been used. Thus, of the 6 identifying the use of specific “other” sources, 50% rated them as highly useful. At the other extreme, 9 of the 20 ranked “other” sources as least useful (scoring at 6–8), 2 indicating using information shared from other workplaces; however, 7 indicating no other source was actually used. Among all 20, the most common other sources used (listed from most to least frequent) could be classified as:

1. Local, regional, or national broadcast news [22%];
2. Other workplace policies (e.g., local hospital, other industry) [18%];
3. Regional health authority [18%];
4. Internet websites (government agencies, social media) [18%];
5. Local healthcare professional (e.g., doctor, nurse, health & safety specialist) [15%];
6. Communication with neighboring SAR teams [4%] or an epidemiologist on staff [4%].

Only one instance was sharing information between local stations mentioned, and there was no indication of BCSARA or RCMSAR headquarters enlisting advice from the few infectious diseases epidemiologists identified by local stations.

Regarding COVID-19 impacting operational readiness, constraint of training was a common refrain. Several mentioned this delaying attainment of advanced specialization certification and making it more difficult to maintain communication, morale, proficiency, and/or recruitment. Some used virtual modalities when in-person training was prohibited; while some virtual sessions may have been recorded for future use, there was no indication of sharing archived locally produced resources outside of individual stations. Fifty-four per cent of respondents reported no loss of active members due to pandemic concerns; others reported from 2% to 25% loss due to individual concerns about personal risk, someone contracting COVID-19, or, in a few cases, refusing to divulge vaccination status or frankly expressing anti-vaccine and anti-masking behavior. In some, this resulted in losses among the limited number of leadership-level team members. Dedicated volunteers enabled the vast majority of local stations to remain available for all emergency taskings throughout the pandemic, although some noted having to respond with fewer than the usual size crew on SAR missions. Only 12.1% had to stand down at times, being unavailable for periods ranging from 1 day (2 stations), a couple of weeks (one station), to months (one station).

Presuming our respondents are representative of all BC’s local volunteer SAR stations, these estimates can be made for percentages among all 109 local stations across the province (95% confidence interval calculated by Poisson^5^ or binomial approximation, with Finite Population Correction Factor^3^ applied):

- Able to remain in service without interruption: 79%–97%
- Having PPE including eye protection (goggles or visors): 67%–90%
- Having PPE including skin protection (disposable gloves): 92%–100%
- Having PPE including respiratory protection (disposable masks): 92%–100%
- Using PPE in a manner consistent with professional counterparts: 0%–13% (due to inconsistencies in eye protection; recognition of aerosol-generating procedure as reason to switch from surgical to N95 mask; replacement of latex with nitrile gloves).

### Comparison of BCSARA, RCMSAR, & professional first responder update documents

On 11 March 2020 BCSARA and on 12 March 2020 RCMSAR initially alerted their members. Within a few days, the 2 organizations diverged on information sources (both relying on provincial websites oriented for the general public, RCMSAR also taking Canadian Coast Guard circulars, only BCSARA citing the provincial update website dedicated to keeping emergency first responders informed). BCSARA and RCMSAR also diverged in emphasis (one quicker to prohibit large or non-essential gatherings; one quicker to encourage stockpiling essential supplies in recognition of coming supply chain disruptions; etc.). One organization seemed, early in 2020, to mandate wearing a mask when in close face-to-face contact (as in rendering first aid) as standard operating procedure while the other encouraged mask usage when social distancing cannot be maintained but initially stipulated that it remained a personal choice (later changing usage to a station-level choice until months later changing it to a headquarters-level universal requirement for all personnel). National shortages of medical grade masks prompted hospitals and ambulance services to develop instructions for safe storage and individual reuse of disposable N95 masks by their professionals; conversely, volunteer SAR members received instruction to not reuse masks. As mutant virus variants of concern arose and understanding of transmission dynamics grew, periodic infection prevention advisory updates were issued by federal and provincial officials in public health and other government agencies. Nongovernmental organizations, like BCSARA and RCMSAR, contextualized these updates in notices for their members. British Columbia Search and Rescue Association and RCMSAR’s updates differ in format. British Columbia Search and Rescue Association each address 1 specific topic (e.g., mask use) or audience (e.g. incident commander); RCMSAR duplicate the previous comprehensive coverage of all topics, with section headings indicating date of an update to that individual section. Table 6 summarizes the crux of all these updates in the order they were issued.

**Table 6.** Timeline of infection prevention guidance document revisions and updates

Year	BCSARA	RCMSAR
	(Day/Month issued)	(Day/Month issued)
2020	*11 March:* *Risk Recognition*: BCSARA Health & Safety Committee monitoring the situation *Risk Mitigation*: No change to usual hygiene measures *Hypertext links*: https://www2.gov.bc.ca/gov/content/health/about-bc-s-health-care-system/office-of-theprovincial-health-officer/current-health-topics/pandemic-influenza & http://www.bccdc.ca/health-info/diseases-conditions/coronavirus-(novel)	*12 March:* *Risk Recognition*: RCMSAR monitoring BCCDC, health experts & Canadian Coast Guard *Risk Mitigation:* No change to meetings training, operations, or other events; if exposed or tested positive notify leadership *Hypertext links:* http://www.bccdc.ca/health-info/diseases-conditions/coronavirus-(novel) & https://www.healthlinkbc.ca/health-feature/coronavirus-disease-covid-19
	*16 March:* *Risk Recognition:* Encourages everyone to follow reasonable steps from Ministry of Health & BCCDC guidance to protect themselves and their group. *Risk Mitigation:* Advised to self-monitor health, use low threshold of suspicion, and be proactive about ordering cleaning, sanitization, or medical supplies. Training at discretion of group leaders; social distancing (1 m); and staying home if any symptoms of COVID-19 *Hypertext links:* http://www.bccdc.ca/health-info/diseases-conditions/covid-19 & https://www2.gov.bc.ca/gov/content/health/about-bc-shealth-care-system/office-of-the-provincial-health-officer/current-health-topics/pandemic-influenza & http://www.bccdc.ca/healthprofessionals/clinical-resources/covid-19-care/clinical-care/first-responders	*13 March:* *Risk Recognition:* Increased risk from travel & large groups recognized *Risk Mitigation:* No gatherings >25 persons, no non-critical gatherings; every person to have own PPE; use of gloves & bleach solution to clean is specified *Hypertext links:* none
	*20 March* *Risk Recognition:* Addresses questions from SAR stations due to small crew space inside helicopters. *Risk Mitigation:* Eight bullet point items for planning helicopter operations emphasize screening, social distancing, and avoiding excess crewing. Lists 11 items to “sterilize” between flights. *Hypertext links:* Highlights BCSARA resource page & provincial self-screening questions website.	*17 March* *Risk Recognition:* Increased exposure risk from travel & large groups, importance of appropriate surface cleaning products & techniques. *Risk Mitigation:* Prohibits face-to-face activities other than rescue taskings & essential maintenance; 70% alcohol added as alternative to bleach. *Hypertext links:* http://www.bccdc.ca/health-info/diseases-conditions/covid-19
	*23 March* *Risk Recognition:* Advises that sick people are less likely to hike, so team members are just as great a risk as subjects. Lists fever, cough, shortness of breath, sore throat, fatigue, or known exposure as screening criteria. *Risk Mitigation:* Notes that PPE can be problematic in SAR environment; describes how to don & doff. Emphasizes social distancing as primary consideration in planning any kind of activity. Detailed guidelines developed by a licensed advanced care paramedic cite several BCCDC websites (variously intended for public, healthcare professionals, and emergency preparedness) along with a BCSARA resource website. *Hypertext links:* http://www.bccdc.ca/health-professionals/clinical-resources/covid-19-care/infection-control & https://www2.gov.bc.ca/gov/content/covid-19/info/response & http://www.bccdc.ca/ & other pages inside BCSARA Members Only website section	*26 March* *Risk Recognition:* Unchanged. *Risk Mitigation:* Advises to wear gloves at all time, remove & dispose of them properly (“as taught during FA”). Advises how to clean vessel & equipment, and to consider refraining from using electronic communication systems in helmets if there has been suspected or confirmed case within station. Reiterates ban on non-essential travel, training, face-to-face meetings, special & social events will continue indefinitely. Advises against boarding other vessels unless necessary to save a life. *Hypertext links:* www.cdc.gov/coronavirus/2019-ncov/specific-groups/people-at-higher-risk.html & www.bccdc.ca/health-info/diseases-conditions/covid-19
	*25 March* *Risk Recognition:* “Guidance for GSAR groups having difficulty accessing minimum PPE” advises widespread shortage of N95 masks, gloves, & safety glasses have caused several groups to stand down until they could access supply. *Risk Mitigation:* Reminds that situation is “complex and constantly changing”; do not take unnecessary risks; self & team safety is paramount; do not go out into the field unless equipped with all PPE. *Hypertext links:* Provides email address for the SAR Volunteer Joint Health & Safety Committee to contact with any health or safety questions.	*29 April* *Risk Recognition:* Unchanged. *Risk Mitigation:* Reiterates points in previous update. Advises “If potential exposure exists, N95 or KN95 masks, if available, must be used on patients with suspected COVID 19 symptoms, and by crews when managing a patient. Otherwise, wearing of surgical masks will suffice for both crew and patients to help mitigate potential virus transfer.” “Do not reuse masks” “strongly encourage members to wear a mask if available and practical during operations where social distancing is not achievable. Wearing of masks, however, remains a personal choice and should not impede safe or effective operation” *Hypertext links:* www.bccdc.ca/health-info/diseases-conditions/covid-19/prevention-risks/masks
	*28 March* *Risk Recognition:* Reviews modes of transmission; answers volunteers’ request for guidance on use of masks for respiratory protection. *Risk Mitigation:* Notes that BCSARA follows provincial guidance for health care workers & paramedics; gives specific advice on when who should wear masks, why N95, how to don & doff; discusses fit testing. *Hypertext links:* Cites BCCDC websites, BCSARA pages, and a YouTube video on how to don & doff an N95 mask.	*13 May* *Risk Recognition:* Unchanged. *Risk Mitigation:* Reiterates previous instructions but allows face-to-face groups of 5 or less for maintenance, inspections and training to resume as of 15 May. *Hypertext links:* http://www.bccdc.ca/health-info/diseases-conditions/covid-19/prevention-risks/masks & https://www.cdc.gov/coronavirus/2019-ncov/specific-groups/people-at-higher-risk.html & https://www.healthlinkbc.ca/more/health-features/coronavirus-disease-covid-19 & http://www.bccdc.ca/health-info/diseases-conditions/covid-19
	*2 April* *Risk Recognition:* Addresses questions concerning unified joint command to deal with requesting agency, resource members, convergent volunteers, or family members at a scene. *Risk Mitigation:* Thirteen bullet point considerations to enforce screening & social distancing for incident command post *Hypertext links:* none	*19 June* *Risk Recognition:* Unchanged. *Risk Mitigation:* Essentially same message as in previous updates, but HQ courses can resume in July, RHIOT in September; SARExs still on hold. Still advises to not reuse masks. Indicates mask use is a “station/personal” choice, if used must be N95 if available for suspect COVID (otherwise use surgical mask) *Hypertext links:* www.bccdc.ca/health-info/diseases-conditions/covid-19/prevention-risks/masks & www.cdc.gov/coronavirus/2019-ncov/specific-groups/people-at-higher-risk.html
	*9 April* *Risk Recognition*: Specific advice for on-scene incident commander for preplanning, activation, operations, and demobilization phases. *Risk Mitigation:* Links to RADeMS calculator. (BCSARA-developed tool to assist SAR Managers with risk assessment specific to infectious disease, including COVID-19) within members-only portion of BCSARA website; also to the provincial self-assessment tool website. *Hypertext links:* https://www2.gov.bc.ca/gov/content/safety/emergency-management/emergency-management/policies, https://covidcheck.gov.bc.ca/, and website pages within BCSARA members-only site.	*13 August* *Risk Recognition:* Essentially same as previous update. *Risk Mitigation:* Adds new section restricting taking guests on vessels to operational requirements. Now indicates mask use is a “station” choice *Hypertext links:* http://www.bccdc.ca/health-info/diseases-conditions/covid-19/prevention-risks/masks, https://www.cdc.gov/coronavirus/2019-ncov/specific-groups/people-at-higher-risk.html, https://www.healthlinkbc.ca/more/health-features/coronavirus-disease-covid-19 & http://www.bccdc.ca/health-info/diseases-conditions/covid-19
	*28 April* *Risk Recognition:* Lists best practices for cleaning and/or sanitizing rescue equipment, response vehicles, SAR halls, and electronic equipment. *Risk Mitigation:* Emphasizes initial cleaning, and use of alcohol as disinfectant. *Hypertext links:* Links to numerous manufacturers’ sites for instructions concerning specialized items (rope, radios), & comprehensive provincial SAR operating guidelines at https://www2.gov.bc.ca/assets/gov/public-safety-and-emergency-services/emergency-preparedness-response-recovery/embc/volunteers/sar_safety_program_operating_guidelines.pdf	*23 October* *Risk Recognition:* Essentially same as previous update. *Risk Mitigation:* Adds that taking guests on board also requires HQ approval *Hypertext links:* http://www.bccdc.ca/health-info/diseases-conditions/covid-19/prevention-risks/masks, https://www.cdc.gov/coronavirus/2019-ncov/specific-groups/people-at-higher-risk.html, https://www.healthlinkbc.ca/more/health-features/coronavirus-disease-covid-19 & http://www.bccdc.ca/health-info/diseases-conditions/covid-19
	*12 May* *Risk Recognition:* Updates mask advisory as most groups had N95 or no masks previously but now have both surgical & N95. *Risk Mitigation:* Indicates need to wear mask only if >2 m distance cannot be maintained; if social distancing cannot be maintained then instructs to use surgical mask unless N95 if COVID-19 symptoms present. *Hypertext links:* https://bc.thrive.health/covid19/en & http://www.bccdc.ca/health-info/diseases-conditions/covid-19/prevention-risks/masks & https://www.canada.ca/en/public-health/services/diseases/2019-novel-coronavirus-infection/health-professionals/assumptions.html (latter concerning recognition of spread from asymptomatic infections).	*8 November* *Risk Recognition:* Contains same sections as previous update but responds to recent (7 November) public-health orders after surge in COVID-19 cases in Lower Mainland & Fraser Valley *Risk Mitigation:* Ban on non-essential travel, training, face-to-face meetings, special events & social events reinstated in two health regions (Fraser & Vancouver Coastal Health regions) *Hypertext links:* same set here as in previous update, and same set in all following RCMSAR updates.
	*21 May* *Risk Recognition & Mitigation:* Puts into a flowchart format all previous assessment & infection control instructions *Hypertext links*: (none)	*19 November* *Risk Recognition:* Contains same sections as previous update. *Risk Mitigation:* Extends ban to all health regions, cancels HQ & RHIOT training until 2021, changes masking decision from station level to now require everyone on missions or during maintenance. Hypertext links: same as above.
	*10 June* Reviews concept of GSAR risk assessment, adds to other risks the COVID concerns, provides an example to illustrate use of RADeMS calculator to assess activity plus pandemic risk. *Hypertext links:* Internal BCSARA members website pages.	*10 December* Essential training can recommence, but other restrictions remain in place.
	*19 June* Explains acceptable clothing as PPE since medical gowns are not practical. References washing instructions *10 November* Responds to new (7 November) public-health orders after surge in COVID cases in Lower Mainland & Fraser Valley *20 November* Advises that PHO order now extends to all areas of the province. *2 December* Notes that more large scale operations occurred in 2020; provides practical advice for best practices in managing large groups of volunteers.	
	*11 December* Explains 7 principles with illustrative practices to define what constitutes essential training. Advises that travel & activity restrictions be observed by members everywhere, not just in the two geographic locations identified in the public health officer’s new orders.	
2021	*10 January* Announces in accordance with health officer orders of 7 January extending prior orders to February that all requirements in BCSARA updates #14, 15, & 17 continue to be in effect.	*11 January* Notes health officer extension of prior orders; permits resumption of essential training in addition to SAR taskings, essential maintenance and vessel refueling. Limits crew size to 5 for training; continues all other previous requirements
	*28 January* Answers 5 questions stemming from actual exposure situations and how they relate to returning volunteers to SAR activities. *6 February* As with 10 January update, extends required precautions until further notice in accordance with 5 February health officer’s extension announcement. *23 March* Interprets 12 March health officer slight easing of social gathering limitations; notes some have misinterpreted this, advises that regular SAR training is not a social event and not essential training, so remains subject to earlier restrictions. *15 May* Gives status quo summary of the COVID-19 situation, advising that “now is not the time to ease up on restrictions” 27 May Based on 25 May BC Provincial restart plan, provides a 4-phase comprehensive SAR activities restart plan for May through September *24 August* Pauses the restart at phase 3 due to Delta variant surge. SAR restart chart was modified to renew mask & social distance emphasis; also now recommends 2 rather than just 1 vaccination dose. *10 September* Requires proof of being fully vaccinated for indoor group activities of more than 50 people. Defines fully vaccinated as 2 doses; recommends all members become fully vaccinated, but notes Ministry of Health currently “does not perceive a need to require Public Safety Lifeline volunteers to be vaccinated” to participate in training, operations, or administrative activities. *23 December* Pauses restart plan, reimposes stricter mask and other precautions; strongly recommends proof of full vaccination for all indoor activities.	*5 February* Permits resumption of Junior Program training subject to following 11 specific requirements. Otherwise identical to previous updates. *25 March* Permits resumption of conducting pleasure craft safety checks subject to 7 specific requirements. Otherwise identical to previous updates. *25 May* Permits resumption of travel within but not across health regions; expansion of shore training (up to 10 people) & interagency on the water training, (permission of HQ required for anything larger); and pending resumption of HQ courses & RHIOT course in coming months. *15 June* Based on 15 June health officer announcement, now permits resumption of most operations (except guest rides, large-scale meetings & exercises, and fundraising & community events). Removes prior restrictions on group numbers for on water & shore training. Permits inter-unit RCMSAR training; still requires self-screening, mask use & social distancing precautions *1 July* Further eases restrictions (to permit within certain limits guest rides, participation in community & fundraising events); lists 6 specific situations in which masks are required (otherwise being recommended but a station-level decision). *25 August* In accordance with provincial restart plan Phase 3, reinstates more use of masks (in all vessels, indoor spaces, and exposure to public rather than only when social distancing cannot be maintained). *13 September* Adds requirement to show proof of vaccination (at least one dose, effective 13 September; at least 2 doses, effective 24 October) for face-to-face activity participation. Otherwise identical. *26 November* Expands list of permitted activities; still retains all other recent requirements. Changes vessel mask requirement to indoor cabin space rather than all vessels. *21 December* Suspends 10 non-essential activities until 18 January.
2022	*17 February* Notes recent & only inferred transmission during a SAR incident, and recent health officer announcement of easing restrictions. Presents revision to phase 3 of restart chart.	*7 January* Due to Omicron variant case surge, introduces temporary minimum crewing requirements.
	*16 March* Per health officer announcing end of restrictions, modified restart chart now has masking as a personal choice (but still requires proof of full vaccination, use of self-assessment tool, limited number of people where possible, and sanitising high touch surfaces).	*1 February* Permits resumption of several activities, cancels temporary minimum crewing requirement restrictions
	*8 April* Responds to health officer lifting the remaining COVID-19 precautions & presents final modification to SAR restart chart for phase 3.	*17 February* Further eases restrictions on numbers and activities.
		*15 March* Changes mask use to required in 4 specific situations, otherwise reverts to a personal choice.
		*8 April* Reduces required mask use to 2 situations, otherwise a personal choice.

On 13 February 2020, the Canadian Coast Guard instructed its crew members when boarding vessels in response to medical distress to wear an N95 mask (and implies fit testing) plus place a surgical mask on the patient among airborne & contact precautions to be taken.^6^ On 19 March 2020, because of COVID-19 concerns, BC licensed paramedics were instructed to wear “airborne PPE” including N95 mask and face shield when performing cardiopulmonary resuscitation (CPR) and cover the patient’s face with a surgical mask or viral-filter-equipped airway management device. On 22 March, they were cautioned to “use the lowest oxygen flow rate possible to achieve a SpO_2_ of 90” and on 23 March 2020, their emergency call takers were instructed to “advise the public to provide hands-only CPR for adults in cardiac arrest and to cover the patient’s mouth and face.”^7^ On 1 April, professional first responders were instructed to “perform compression-only CPR and defibrillation as necessary with no oxygenation or airway management until paramedics arrive” (memo from BC Emergency Health Services Chief Medical Officer to The Fire Chiefs Association of BC, https://fcabc.ca/index.php/covid-homepage). Volunteer SAR first responders received no such advice from their organizations to change from full CPR to chest compression only (likely because those organizations assumed their contracted first-aid training providers would intercede).

Neither BCSARA nor RCMSAR volunteers were designated with official emergency first responder status by the provincial government (personal communications, BCSARA, & RCMSAR). There was no communication or requests for information between these 2 volunteer SAR organizations and the Provincial Infection Control Network of BC (personal communications, PICNet). Thus, neither had official direct connections to channels of information that regularly provide infection control advice to the province’s professional first responders and healthcare professionals.

## Discussion

COVID-19 literature specific to SAR is not readily found through the usual literature indices. Koester reports finding pertinent results through Google Scholar but zero results searching PubMed, Ovid, and Web of Science.^8^ Koester’s literature review documents similarities and differences among half a dozen SAR infection prevention guidelines from various agencies around the world for maritime, coastal, ground, urban, mountain, and cave rescue. Studies in several countries indicate frequency of rescue calls before versus during the pandemic did not change significantly. However, we are not aware of any previous studies reporting extent to which SAR teams were able to comply with infection prevention guidelines.

Infections caused by SARS-CoV-2 were first detected in Wuhan China late in 2019. In March 2020, the World Health Organization (WHO) declared a pandemic. Beyond infection precaution advisory documents issued by WHO and National Centers for Disease Control (from which SAR organizations can draw general advice), 2 international SAR associations issued their own guidelines specific to SAR operations. In April 2020, the International Maritime Rescue Federation released a comprehensive document,^9^ and in March 2021, the International Search and Rescue Advisory Group released its guidance for urban search and rescue.^10^

Early in 2020, North American public messaging about recognizing COVID-19 cases focused on fever, cough plus recent contact through China travel.^6^ About that same time, postings from clinicians in Britain on the UK-based international Evidence-Based Health discussion list (https://www.jiscmail.ac.uk/cgi-bin/webadmin?A0=evidence-based-health) described loss of sense of smell and taste as the prominent (sometimes exclusive) signs or symptoms in numerous cases already occurring there. An Italian mountain rescue group listed “Difficulty perceiving smells and flavors” in its 2020 checklist.^11^ Also, molecular epidemiology revealed that early SARS-CoV-2 isolates in New York predominately arose via Europe rather than Asia travel.^12^ Thus, from the outset, risk recognition within the biomedical community differed from risk recognition in North America’s more public entities including SAR. Screening criteria used by SAR for risk recognition initially may have been too narrow.

Fortunately, in terms of risk mitigation, BC experienced among the lowest infection and mortality rates in the country and world throughout this pandemic (https://bccdc.shinyapps.io/covid19_global_epi_app/). This was, in no small part, due to targeted evolving precautionary measures introduced by a highly experienced Provincial Health Officer working closely with a prudent Minister of Health.^13^ Fewer initial cases in many parts of BC reduced relative exposure potential, and personal behavior precautions introduced (viz. social distancing, masking, hand hygiene, encouragement to not work while ill) increased personal safety for the general population and its workforces. Evidence-informed periodic updates on the pandemic and its implications for precautionary measures were provided through daily television presentations by the Provincial Health Officer and Minister of Health; an information dashboard and weekly situation reports posted on government website (http://www.bccdc.ca/health-info/diseases-conditions/covid-19); popular news outlets frequently reflecting on the most recent briefings; guidance documents periodically issued by professional and other organizations and unofficial advice offered by various academic medicine specialists. Less trustworthy misinformation and opinion also abounded on social media. British Columbia Search and Rescue Association and RCMSAR based timely update releases on contextualizing reputable health department website information, and this study confirms their perceived value.

Several opportunities for improvement are evident from our exploratory study. First, most local volunteer SAR stations’ criterion for selecting an N95 versus surgical mask for respiratory protection has not been consistent with the longstanding risk recognition and mitigation standard in hospitals that also was adopted by ambulance service paramedics. Second, why local stations have or need both latex and nitrile gloves is not clear. Third, among thousands of SAR volunteers, some local station members have first-hand experience with SAR operations as well as occupational expertise in chemical, biological, radiation, and/or nuclear hazards. Those would be particularly valuable on health and safety or first-aid committees, but neither BCSARA nor RCMSAR appears to have inventoried these qualifications at national or regional levels so may want to consider recruiting such members onto higher-level committees. Fourth, there appears to have been no effort on either side to create information linkages between the volunteer SAR first-aid setting and the provincial network for infection control professionals (PICNet). Provincial network for infection control professionals explained to us “PICNet’s purview is to provide infection prevention and control guidance and support for health care settings, therefore, it would be outside of PICNet’s scope to provide recommendations or guidance in non-healthcare settings.” Infection control experts do provide advice for emergency health services professional responders employed in hospital emergency departments, and to ensure continuity to some extend that advice for ambulance service attendants who stabilize and transport patients to hospital. Some, as a professional courtesy, also occasionally provide police or firefighter groups with pertinent advice on infection risks and prevention practices. Infection control professionals should remember there are volunteer counterparts who could benefit from the same information provided to professional first responders. Fifth, a definitive review, documenting only 15 infections in 30 years, transmitted from administering CPR to millions, stated in 1998: “Recent research suggests that chest compression is more important in achieving adequate ventilation than mouth-to-mouth ventilation alone; in the future, rescue breathing may not be considered necessary for basic cardiac life support…”^14^ More recent literature confirms compression-only CPR’s effectiveness.^15^ The UK adopted compression-only CPR for volunteer SAR nationally.^16^ Since not everyone on search missions is likely to have at hand a bag-valve-mask (aka Ambu bag) fitted with virus filter, nor have maintained proficiency in its use, and probability of favorable outcome drops precipitously every minute high-quality CPR is delayed, compression-only CPR is a more-practical less-expensive SAR response. When supplemental oxygen arrives, either nonrebreather mask or bag-valve-mask can then be placed by someone wearing an N95 mask while administering high-flow rate oxygen.^9,10^

This exploratory study has several limitations. First, although we assume value rankings received from local stations are accurate regarding sources of information available to guide leadership decisions, we only requested participation of station leaders so did not poll all station members to confirm these are consensus opinions. Second, while it is likely that stations claiming to have both types of masks and gloves have them concurrently, given our cross-sectional research design, it is possible that stations had only one type at a time sequentially. Third, geographic and size distribution of participating stations align well with the sampling frame (all stations) so can be representative; however, we have no way within the study to quantitatively assess extent of any non-response bias.

## Supporting information

Birnbaum et al. supplementary material Birnbaum et al. supplementary material

## Supplementary material

For supplementary material accompanying this paper visit http://doi.org/10.1017/ash.2023.463. click here to view supplementary material

## Note

This manuscript is a condensed version of a longer report that the authors provided to BCSARA and RCMSAR for their own internal use.

## References

[1] British Columbia Academic Health Sciences Network. BC COVID-19 Research & Collaboration Symposium, Vancouver BC, 2020. https://www.bcahsn.ca/learning/find-resource/bc-covid-19-research-collaboration-symposium-recordings. Accessed March 10, 2022.

[2] Klompas M , Baker M , Rhee C. What is an aerosol-generating procedure? JAMA Surg 2021;156:113–114. doi: 10.1001/jamasurg.2020.6643 33320188

[3] Anderson DR , Sweeney DJ , Williams TA , Statistics for Business and Economics, 8th edition. Cincinnati, Ohio: South-Western/Thomson Learning; 2002, pp. 257 (FPC) & 495 (Bonferroni).

[4] Remington RD , Schork MA , Statistics with Applications to the Biological and Health Sciences, 2nd edition. Englewood Cliffs NJ: Prentice Hall Inc.; 1970, pp. 315.

[5] Hanley JA , Lippman-Hand A , If nothing goes wrong, is everything all right- interpreting zero numerators, JAMA 1983;249:1743–1745 6827763

[6] Government of Canada. Canadian coast guard operations safety bulletin OSB 05-20; 2020. https://www.ccg-gcc.gc.ca/publications/safety-bulletin-securite/5404-2020-05-eng.html. Accessed May 16, 2022.

[7] BC Emergency Health Services. COVID-19 clinical practice changes summary; 2020. https://handbook.bcehs.ca/clinical-resources/covid-19/covid-19-summary-of-clinical-practice-changes/. Accessed March 14, 2022.

[8] Koester RJ , Review of search and rescue response guidelines to COVID-19. J Search Rescue 2020;4:214–234.

[9] IMRF. IMRF COVID-19 operational guidelines; 2020. https://www.international-maritime-rescue.org/Handlers/Download.ashx?IDMF=77b41c9f-1553-4e69-b6bb-f2dcb1b1c729. Accessed May 16, 2022.

[10] INSARAG. INSARAG Medical Working Group COVID-19 deployment technical guidance note; 2021. https://www.insarag.org/wp-content/uploads/2021/03/MWG-Covid-19-USAR-Response-Technical-Guidance-Note-Final.pdf. Accessed May 16, 2022.

[11] Massullo D , Fiorella S , Rubcich P , Romano D , Facchetti G. Mountain rescue during the COVID-19 outbreak: considerations and practical implications. Wilderness Environ Med 2021;32:123–125. 33317931 10.1016/j.wem.2020.09.003 PMC7510558

[12] Gonzalez-Reiche AS , Hernandez MM , Sullivan M , et al. Introductions and early spread of SARS-CoV-2 in the New York City area. Science 2020;369:297–301. 10.1126/science.abc1917. 32471856 PMC7259823

[13] de Faye B , Perrin D , Trumpy C. COVID-19 lessons learned review: final report; 2022. https://www2.gov.bc.ca/assets/gov/public-safety-and-emergency-services/emergency-preparedness-response-recovery/embc/reports/covid-19_lessons_learned_report.pdf. Accessed March 23, 2023.

[14] Mejicano GC , Maki DG. Infections acquired during cardiopulmonary resuscitation: estimating the risk and defining strategies for prevention. Ann Intern Med 1998;129;813–828. 9841588 10.7326/0003-4819-129-10-199811150-00014

[15] Zhan L , Yang LJ , Huang Y , He Q , Liu GJ. Continuous chest compression versus interrupted chest compression for cardiopulmonary resuscitation of non-asphyxial out-of-hospital cardiac arrest. Cochrane Datab Syst Rev 2017. 10.1002/14651858.CD010134.pub2. PMC6464160 28349529

[16] Kitchen WR. Masks in the mountains: how a UK search and rescue team prepared for COVID-19. Emerg Med J 2021;38:248. 33355271 10.1136/emermed-2020-210580

