# Supplementary material for: COVID-19 and British Columbia’s volunteer search and rescue workers: risk recognition and risk mitigation
Source: Antimicrob Steward Healthc Epidemiol. 2023 Nov 3;3(1):e195. doi: 10.1017/ash.2023.463 (PMC10654942; doi:10.1017/ash.2023.463)
Supplement: Birnbaum et al. supplementary material [file S2732494X23004631sup001.docx]

PLEASE ANSWER THE FOLLOWING 7 QUESTIONS:

1. Since January 2020, which approach or approaches has your own local group/station/unit used to stay abreast of the evolving COVID19 situation? Please indicate whether monitored (yes or no) as well as how often, and how often the information derived was shared with all your members (e.g., as a periodic summary) – possible entries in the last column include irregularly, quarterly, monthly, weekly. Please enter a response for every line in this table.

| *Information Source Monitored* | *Yes* | *No* | *If Yes How Often Used* | *How Often Shared?* |
| --- | --- | --- | --- | --- |
| Receive updates mailed from RCMSAR or BCSARA |  |  |  |  |
| Monitor postings on the RCMSAR or BCSARA member website |  |  |  |  |
| Monitor PHAC federal COVID19 Website |  |  |  |  |
| Monitor BCCDC COVID19 Website Dashboard |  |  |  |  |
| Monitor BCCDC COVID19 Website Weekly Situation Reports |  |  |  |  |
| Watch BC Provincial Health Officer daily briefings |  |  |  |  |
| Receive updates from local health official(s) or local health agencies |  |  |  |  |
| Other sources (please specify) |  |  |  |  |

2. From most to least useful to you, please rank these sources of information (1=most, 8=least useful).

| *Information Source* | *1-8* |
| --- | --- |
| Receive updates mailed from RCMSAR or BCSARA |  |
| Monitor postings on the RCMSAR or BCSARA member website |  |
| Monitor PHAC federal COVID19 Website |  |
| Monitor BCCDC COVID19 Website Dashboard |  |
| Monitor BCCDC COVID19 Website Weekly Situation Reports |  |
| Watch BC Provincial Health Officer daily briefings |  |
| Receive updates from local health official(s) or local health agencies |  |
| Other sources (please specify) |  |

Briefly, what is it about the most useful items that make them the most useful, and the least useful the least useful?

3. What items of personal protective attire do your members have available for use during first aid rescue situations, and how were they obtained. On each line, please place an X or check-mark in the second column if used, and in one or more of the next columns to indicate source.

| *Item* | *√* | *Donated* | *HQ Supplied* | *Local purchase* | *Member supplies own* |
| --- | --- | --- | --- | --- | --- |
| Face shield or goggle |  |  |  |  |  |
| Surgical mask |  |  |  |  |  |
| N95 respirator mask |  |  |  |  |  |
| Nitrile gloves |  |  |  |  |  |
| Latex gloves |  |  |  |  |  |
| Hand sanitizer |  |  |  |  |  |
| Surface disinfectant |  |  |  |  |  |

If both surgical and N95 masks are made available, on what basis do individuals decide whether to use a surgical mask or an N95 mask?

4. How many SAR volunteers are normally the active members of your local group/station/unit?

5. On average, what percentage of your volunteer active members have withdrawn from being on call because of their concern about being at risk from COVID19 exposure?

6. To what extent and in what way(s) has COVID19 impaired your local group/station/unit’s operation readiness? Were you able to stay on call every week? Since January 2020, how many (if any) weeks did your group/station/unit have to stand down due to on-call staffing shortages?

7. From January through March 2020, news of COVID19 started circulating widely in North America. Infection prevention advice evolved since then as more was discovered about the unique newly-emerged virus, its transmission and the diseases it causes. The research team will obtain from BCSARA and RCMSAR headquarters all COVID19 policy or procedure document advisories they sent to your local group/station/unit. However, we believe that some groups/stations/units received COVID19 update advice from other sources as well. Please list the source and frequency (and attach if possible) any such additional COVID19 infection prevention measures advisory documents your station received from other sources and used to protect your volunteer members.
